# Within-Guideline Alcohol Consumption Protects Against Dementia?: Offset Effect of History of Drinking Problems

**DOI:** 10.1093/geroni/igaa057.3309

**Published:** 2020-12-16

**Authors:** Penny Brennan

**Affiliations:** University of California, San Francisco, San Francisco, California, United States

## Abstract

Research on the prospective relationship between older adults’ alcohol consumption and their subsequent risk of dementia and cognitive impairment, no dementia (CIND) has been limited by inconsistent definitions of “moderate” drinking, use of short follow-ups, and an exclusive focus on either amounts of alcohol, or history of drinking problems, as predictors. To overcome these limitations we analyzed a longitudinal, 18-year Health and Retirement Study cohort (n=4,421) to determine how older adults’ baseline membership in one of six drinking categories (Non-Drinker, Without and With a History of Drinking Problems (HDP); Within-Guideline Drinker, Without and With a HDP; and Outside-Guideline Drinker, Without and With a HDP) predicted dementia and CIND 18 years later. Among participants with No HDP, 12.6% of Non-Drinkers, 5.2% of Within-Guideline Drinkers, and 8.8% of Outside-Guideline Drinkers were classified as having dementia at the 18-year follow-up; among participants With HDP, 14.1% of Non-Drinkers, 8.9 % of Within-Guideline Drinkers, and 6.9% of Outside-Guideline Drinkers were classified with dementia. Being a baseline Within-Guideline Drinker with No HDP reduced the likelihood of dementia 18 years later by 45%, independent of baseline demographic and health characteristics; being a baseline Within-Guideline Drinker With a HDP reduced the likelihood of dementia by only 13% (n.s.). Similar patterns obtained for the effects of baseline drinking group membership on likelihood of CIND at follow-up. These findings suggest that consuming alcohol at levels within validated guidelines for low-risk drinking may protect against dementia and CIND, but only among older adults who have no history of drinking problems.

